# Early administration of low dose norepinephrine for the prevention of organ dysfunctions in patients with sepsis

**DOI:** 10.1186/2197-425X-3-S1-A417

**Published:** 2015-10-01

**Authors:** MK Arslantas, F Gul, A Kararmaz, F Sungur, HO Ayanoglu, I Cinel

**Affiliations:** Department of Anesthesiology and Reanimation, Marmara University Pendik Education and Research Hospital, Istanbul, Turkey; Department of Anesthesiology and Reanimation, Marmara University School of Medicine, Istanbul, Turkey

## Introduction

The importance of vasopressors in achieving the mean arterial pressure target in the early resuscitation of septic shock has been demonstrated and sepsis has been recently defined as a systemic response to infection with the presence of some degree of organ dysfunction [1, 2]. However, timing of norepinephrine (NE) for the prevention of organ dysfunctions in sepsis has not been investigated.

## Objectives

We studied the role of earlier low dose NE administration as a neurohormone rather than as a vasopressor for the prevention of organ dysfunctions in sepsis.

## Methods

To achieve and maintain MAP at ≥ 70 mm Hg and central venous oxygen saturation ≥ 70%, in Group Early-NE (n = 16), fluid challenge and norepinephrine therapy concurrently was started within 6 hours at a dose of 0.1 µg/kg/min to a maximal dose of 0.3 µg/kg/min in sepsis patients with a decreasing trend in MAP from the baseline. Group Late-NE (n = 19) consisted of the sepsis patients whom hypoperfusion persists in spite of fluid resuscitation, and norepinephrine was started after 6 hours with the dosage like Early-NE. In both groups, the norepinephrine infusion was continued until the resolution of septic shock.

## Results

The average time to initial antimicrobial treatment was not significantly different in both groups. Mortality rate and organ dysfunctions (including cardiovascular, respiratory, coagulatory, renal and liver) were significantly lower in Group Early-NE.

## Conclusions

Our results show that early administration of low dose norepinephrine in patients with sepsis is associated with a decreased organ dysfunctions and an increased survival rate. Although earlier vasopressor therapy can help to trigger the quality of delivered care, more importantly a decrease in net fluid balance seems to be the underlying key factor in our study. Early initiation of NE, as a new approach, should be assessed detailed regarding the pathophysiology of the organ dysfunctions in sepsis.

**Figure 1:**
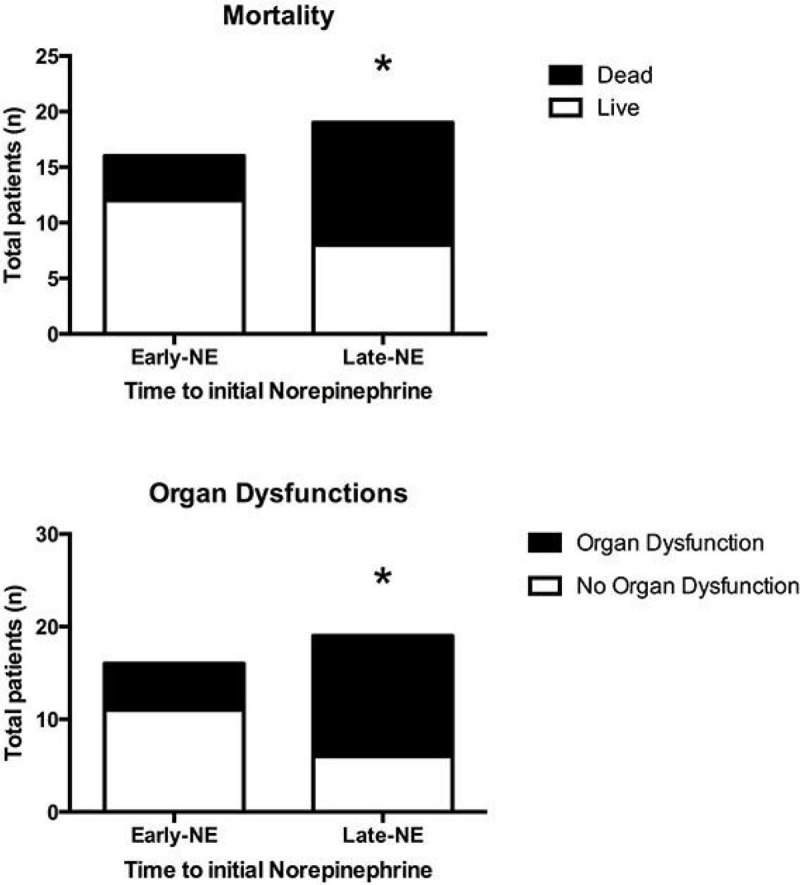
**Accumulated net positive fluid balance at the first 3 day was higher in the Late-NE group (p < 0.05).**

**Figure 2:**
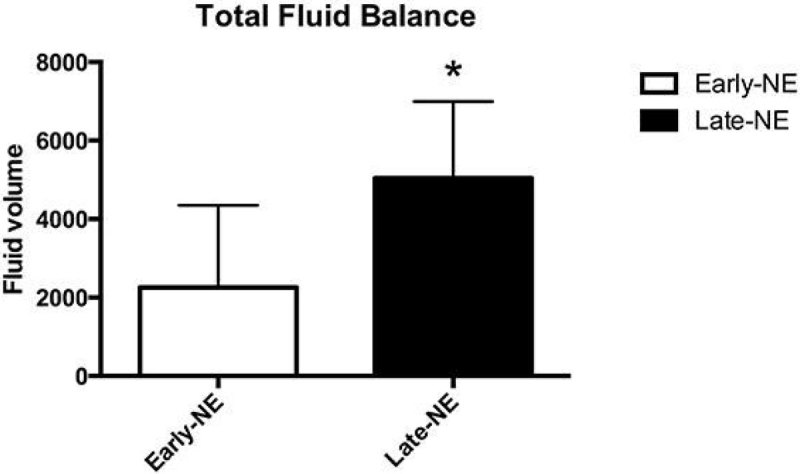
**Urine output at 2nd and 3rd days were significantly higher and serum creatinine levels at 2nd and 3rd days were significantly lower for the Early-NE group compared to the Late-NE groups.**

**Figure:**
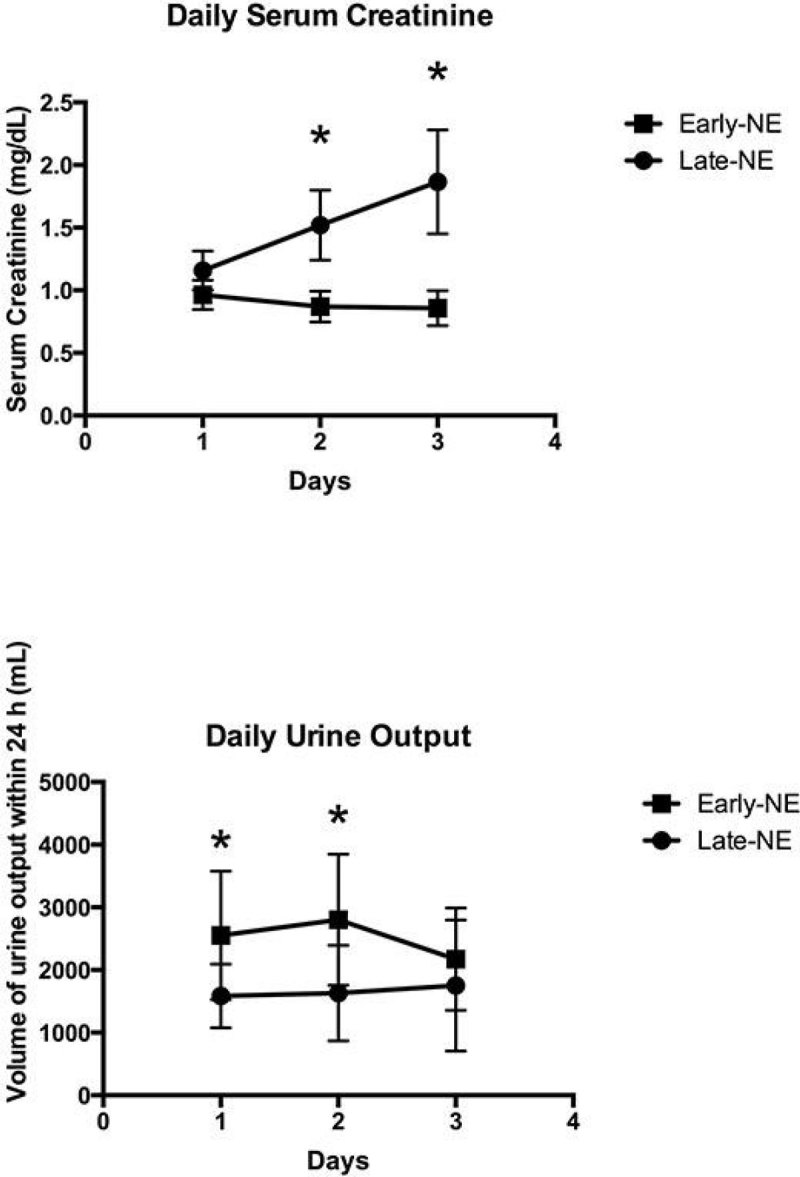
Figure 3
